# Dynamics of HIV-1 Molecular Networks Reveal Effective Control of Large Transmission Clusters in an Area Affected by an Epidemic of Multiple HIV Subtypes

**DOI:** 10.3389/fmicb.2020.604993

**Published:** 2020-11-13

**Authors:** Mingchen Liu, Xiaoxu Han, Bin Zhao, Minghui An, Wei He, Zhen Wang, Yu Qiu, Haibo Ding, Hong Shang

**Affiliations:** ^1^NHC Key Laboratory of AIDS Immunology (China Medical University), National Clinical Research Center for Laboratory Medicine, The First Affiliated Hospital of China Medical University, Shenyang, China; ^2^Units of Medical Laboratory, Chinese Academy of Medical Sciences, Shenyang, China; ^3^Key Laboratory of AIDS Immunology, Chinese Academy of Medical Sciences, Shenyang, China; ^4^Key Laboratory of AIDS Immunology of Liaoning Province, Shenyang, China; ^5^Collaborative Innovation Center for Diagnosis and Treatment of Infectious Diseases, Hangzhou, China

**Keywords:** HIV-1, transmission cluster, phylodynamics, molecular epidemiology, antiretroviral therapy

## Abstract

This study reconstructed molecular networks of human immunodeficiency virus (HIV) transmission history in an area affected by an epidemic of multiple HIV-1 subtypes and assessed the efficacy of strengthened early antiretroviral therapy (ART) and regular interventions in preventing HIV spread. We collected demographic and clinical data of 2221 treatment-naïve HIV-1–infected patients in a long-term cohort in Shenyang, Northeast China, between 2008 and 2016. HIV pol gene sequencing was performed and molecular networks of CRF01_AE, CRF07_BC, and subtype B were inferred using HIV-TRACE with separate optimized genetic distance threshold. We identified 168 clusters containing ≥ 2 cases among CRF01_AE-, CRF07_BC-, and subtype B-infected cases, including 13 large clusters (≥ 10 cases). Individuals in large clusters were characterized by younger age, homosexual behavior, more recent infection, higher CD4 counts, and delayed/no ART (*P* < 0.001). The dynamics of large clusters were estimated by proportional detection rate (PDR), cluster growth predictor, and effective reproductive number (R_*e*_). Most large clusters showed decreased or stable during the study period, indicating that expansion was slowing. The proportion of newly diagnosed cases in large clusters declined from 30 to 8% between 2008 and 2016, coinciding with an increase in early ART within 6 months after diagnosis from 24 to 79%, supporting the effectiveness of strengthened early ART and continuous regular interventions. In conclusion, molecular network analyses can thus be useful for evaluating the efficacy of interventions in epidemics with a complex HIV profile.

## Introduction

The rapid evolution of human immunodeficiency virus (HIV) leaves measurable footprints in the viral genome that can be used for epidemic surveillance by phylogenetic analysis (Hassan et al., 2017). In recent years, a simplified genetic distance-based method has increasingly been used to infer HIV-1 networks in the population, in which a molecular cluster represents a group of individuals infected with genetically similar HIV strains (Smith et al., 2009; US-CDC, 2018; Han et al., 2020). The expansion of molecular clusters represents recent and ongoing HIV transmission, and key subpopulations associated with such clusters are targets for prioritized interventions such as partner tracing and HIV testing through partner services (Green et al., 2017) for diagnosis of unknown HIV-positive cases (Green et al., 2017). Immediate antiretroviral therapy (ART) is recommended for individuals diagnosed with HIV infection (Hoenigl et al., 2016b), along with pre-exposure prophylaxis (PrEP) for socially linked individuals who test negative (Kasaie et al., 2017).

HIV molecular networks can be used not only to guide targeted intervention in key subpopulations (Aldous et al., 2012; Lubelchek et al., 2015; Castley et al., 2017; Chaillon et al., 2017; Zhang et al., 2017; Stecher et al., 2018; Volz et al., 2018; Ragonnet-Cronin et al., 2019; Board et al., 2020), but also to reconstruct the history of HIV spread between populations (Kouyos et al., 2010; Brenner et al., 2017; Patino-Galindo et al., 2017; Pineda-Pena et al., 2018; Delgado et al., 2019; Fabeni et al., 2019). Several local and national studies conducted in recent years have monitored the dynamics of HIV molecular clusters and evaluated their expansion speed (Chan et al., 2015; Mehta et al., 2018; Ragonnet-Cronin et al., 2018; Wertheim et al., 2018; Jovanovic et al., 2019; Dennis et al., 2020). However, most of these studies focused on areas with only subtype B or C HIV-1 epidemics, and few local studies have reconstructed HIV transmission history in an area with multiple HIV subtypes, which may be more complicated because of the variable transmission dynamics and evolution rates of different HIV-1 strains in the population.

China is among the countries with the highest numbers of HIV-1 subtypes in the world outside of West and Central Africa (Li et al., 2016; Hemelaar et al., 2019). According to the latest National HIV Molecular Epidemiological Survey in China, as many as 18 known HIV-1 subtypes and circulating recombinant forms (CRFs) have been detected, with four main subtypes accounting for 90% of total infections: CRF07_BC (41.9%), CRF01_AE (33.2%), CRF08_BC (10.9%), and subtype B (4.0%) (Li et al., 2016). Shenyang, a city in northeastern China, records about 1000 new cases of HIV infection annually, which represents a mid-level HIV incidence in the country (Li et al., 2016). However, Shenyang has experienced multiple–HIV-subtype epidemics, initially in the heterosexual population and later in the homosexual population (Han et al., 2010; Han et al., 2013). Chinese guidelines for ART initiation in HIV-infected patients have been updated several times: in 2002, when China’s free ART program was introduced, it was recommended for the World Health Organization stage III or IV, symptomatic disease, extrapulmonary tuberculosis, or CD4 + T cell counts < 200 cells/μl; this was amended to CD4 + T cell counts ≤ 350 cells/μl in 2008; CD4 + T cell counts ≤ 500 cells/μl in 2014; and finally, to a recommendation of immediate treatment for all diagnosed cases of HIV infection in 2016 (China-CDC, 2005; AIDS-Professional-Group, 2011; AIDS-Professional-Group, 2015; China-CDC, 2018; Xu et al., 2020). In 2008, a large-scale prospective cohort of men who have sex with men (MSM) was established in Shenyang that included thousands of HIV-1–negative MSM who were regularly followed up and screened for HIV infection status through serologic and pooled nucleic acid testing (Xu et al., 2010). The impact of the abovementioned strengthened treatment and intervention policies on the local HIV epidemic has yet to be systematically evaluated.

In this study, we reconstructed the HIV-1 molecular networks of three major subtypes of HIV based on partial pol gene sequences of HIV-1 who were newly diagnosed with HIV infection between 2008 and 2016 in Shenyang. Demographic and clinical data were also analyzed to characterize the cases associated with larger clusters. We then evaluated the expansion dynamics of each large cluster (≥ 10 cases) and assessed the effects of strengthened early ART and regular interventions on the local HIV epidemic.

## Materials and Methods

### Study Population

The study enrolled 2221 individuals with newly diagnosed HIV infection at the First Affiliated Hospital of China Medical University from 2008 to 2016. This hospital is the largest general hospital in Shenyang city, and admits nearly half of all HIV infection cases. All individuals who were diagnosed at or were referred to the hospital for treatment between 2008 and 2016 were included in the study. Blood samples were collected at the time of diagnosis or before ART. Demographic data including sex, age, ethnic group, occupation, education, marital status, HIV risk behaviors, date of diagnosis, date of ART initiation, and resident city; and clinical data including viral load and CD4 + T cell count were collected. The study was approved by the ethics committee of the First Affiliated Hospital of China Medical University. All study participants signed informed consent forms.

### HIV-1 Limiting Antigen (Lag) Avidity Enzyme Immunoassay

Recent HIV infection (RHI) was distinguished from chronic HIV infection (CHI) using the LAg-Avidity EIA kit (Maxim Biomedical, Rockville, MD, United States) according to the manufacturer’s instructions. The normalized optical density (OD) of each sample was calculated as OD of the sample divided by that of the calibrator. RHI was defined as OD ≤ 2.0 in the screening test, and OD ≤ 1.5 in the confirmatory test (Kouyos et al., 2010).

### HIV-1 Sequences and Cluster Identification

RNA extraction and partial pol gene amplification and sequencing were performed as previously described (Zhao et al., 2011). The sequences were aligned using the online HIVAlign program^1^ and manually edited. The retention length was 1015 bp (HXB2: 2253–3267). HIV-1 subtypes were determined by phylogenetic analysis after constructing an approximate maximum likelihood tree using Fast Tree 3.0 (Price et al., 2010), in which subtype N was used as the outgroup, the nucleotide substitution model was GTR + G + I, and support values of the nodes were calculated with a Shimodaira Hasegawa-like test (Liu et al., 2011). HIV-1 molecular networks were constructed using HIV Transmission Cluster Engine (HIV-TRACE) (Kosakovsky Pond et al., 2018)^2^ according to a previously described protocol (Wertheim et al., 2014; Oster et al., 2015; Whiteside et al., 2015; Wertheim et al., 2016). Briefly, all sequences were aligned with a reference HIV-1 pol sequence and the Tamura–Nei 93 pairwise distance was calculated for each pair of sequences. To obtain a high-resolution molecular network, we optimized the genetic distance threshold of three major subtypes to identify the largest number of molecular clusters (Wertheim et al., 2017). Pairwise distances of 0.5%, 0.5%, and 0.7% were used as the optimized genetic thresholds for CRF01_AE, CRF07_BC, and subtype B, respectively (Supplementary Figure S3 and Supplementary Table S1). All codons associated with antiretroviral drug resistance were included in this study. Previous studies have shown that the transmission of drug resistance in Shenyang occurs at a low rate (Zhao et al., 2011; Zhao, 2015). We removed codons associated with antiretroviral drug resistance and found that the results were unchanged (data not shown).

### Relationship Between Large Clusters and Others

Clusters with ≥ 10 cases and between two and nine cases were defined as large and small/medium clusters, respectively. The definition of a large cluster is similar to that used in previous studies (Hughes et al., 2009; Leigh Brown et al., 2011; Patino-Galindo et al., 2017; Lorenzin et al., 2019; Rhee et al., 2019; Dennis et al., 2020). We evaluated demographic features of the study population including the time of diagnosis, sex, risk group, age, ethnic group, resident city, marital status, education, and occupation. HIV risk behaviors were categorized as MSM, heterosexual (hetero), injection drug user (IDU), and other/unknown. The following clinical data were analyzed: CD4 + lymphocyte count, HIV-1 RNA viral load, RHI or CHI, and the time between HIV infection diagnosis and ART initiation. Because the standards of ART initiation were updated in 2008 and 2014, the study period was divided into three 3-year phases: 2008–2010, 2011–2013, and 2014–2016. Early and delay ART were defined as initiated ART within 6 months and above 2 years after diagnosis.

### Proportional Detection Rate, Cluster Growth Predictor, and Effective Reproductive Number (R_*e*_)

To describe the dynamics of a given cluster, three parameters–i.e., PDR, cluster growth predictor, and R_*e*_–were calculated as follows. PDR for a given year (j) was calculated as the cumulative number of cases in the cluster sampled up to and including year j, divided by the cumulative number of cases up to and during the last sampling year (i), per observation time between years j and i. PDR ≥ 2 (i.e., a 2-fold increase in size in 1 year) was considered as a significant change (Dennis et al., 2020).

Cluster growth predictor was calculated as previously described (Wertheim et al., 2018) as the number of newly diagnosed individuals in a given year divided by the square root of cluster size at the end of that year. A declining curve indicated that a given cluster had a very low probability of causing an outbreak.

R_*e*_ of each large cluster (≥ 10 cases) was estimated with the birth–death skyline serial model in BEAST v2.4.2 (Jovanovic et al., 2019; Vasylyeva et al., 2019; Vinken et al., 2019; Dennis et al., 2020). R_*e*_ represents the average number of secondary infections caused by a typical infected individual when only part of the population is susceptible. The value is often used to describe temporal changes of an epidemic in a population, with *R*_*e*_ > 1 and *R*_*e*_ < 1 indicating the growth or decline of the epidemic, respectively.

### Statistical Analysis

Results were analyzed with standard statistical tests. Categoric data were compared with the chi-squared test or Fisher’s exact test using SPSS v20.0 (SPSS Inc, Chicago, IL, United States). *P* < 0.05 indicated a statistically significant difference.

## Results

### The Subtype Profile of Study Population and Molecular Network Characteristics

A total of 2221 individuals who were newly diagnosed with HIV infection between January 2008 and December 2016 were included in the study. Of these cases, 75% (1669/2221) were CRF01_AE, 12% (264/2221) were CRF07_BC, 7% (154/2221) were subtype B, and 6% (134/2221) were other CRFs/unique recombinant forms (URFs) (Supplementary Figures S1, S2). After CRF01_AE, CRF07_BC, and subtype B, the top 5 CRFs were CRF59_01B (*n* = 16), CRF55_01B (*n* = 12), CRF08_BC (*n* = 10), CRF67_01B (*n* = 9), and CRF33_01B (*n* = 6). The prevalence of both CRF01_AE and subtype B gradually declined between 2008 and 2016 (from 81.4 to 67.4% and from 10.5 to 6.2%, respectively), while that of CRF07_BC and other CRFs/URFs increased (from 4.7 to 18.1% and from 3.5 to 8.3%, respectively) (Supplementary Figure S2).

**FIGURE 1:**
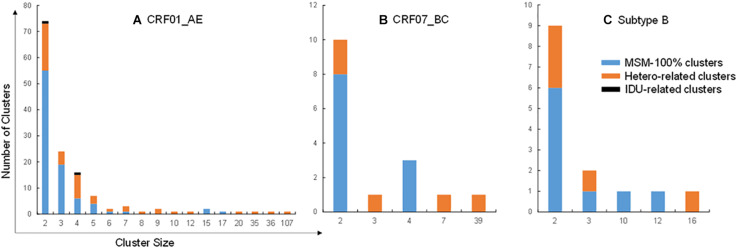
Distribution of risk group clusters. (A–C) Risk group clusters in transmission networks of CRF01_AE **(A)**, CRF07_BC **(B)**, and subtype B **(C)**. Blue, orange, and black columns represents clusters containing 100% MSM and hetero- and IDU-related clusters, respectively.

Molecular networks were constructed for 2087 sequences of CRF01_AE, CRF07_BC, and subtype B using HIV-TRACE. A total of 788 (37.8%) sequences (81.9% CRF01_AE, 10.3% CRF07_BC, and 7.9% subtype B) were linked to at least one other sequence and formed 168 transmission clusters, including 138 of CRF01_AE, 16 of CRF07_BC, and 14 of subtype B, with cluster size ranging from 2 to 107 sequences. Of the 788 clustered sequences, 89.0%, 92.6%, and 88.7% were MSM nodes for CRF01_AE, CRF07_BC, and subtype B, respectively. Of the 168 transmission clusters, 66.1% (111/168) comprised only MSM nodes; 32.7% (55/168) contained hetero nodes, and the percentage of hetero nodes in a hetero-related cluster ranged from 2.6 to 100%. However, 14 hetero-dominated clusters (i.e., in which hetero nodes accounted for more than half of those in the cluster) were all small (cluster size of 2 or 3). Only two clusters of CRF01_AE were IDU-related. Of the 168 clusters, there were 13 large clusters each comprising at least 10 patients, including nine of CRF01_AE clusters, one of CRF07_BC, and three of subtype B (Figure 1).

### Population Characteristics of Large Clusters and Other Groups

Of the 2087 individuals newly diagnosed with HIV infection, 444 (21.3%) and 344 (16.5%) belonged to small/medium (2–9 cases) and large (≥ 10 cases) clusters, respectively, whereas 1299 (62.2%) were non-clustered. We evaluated factors associated with clustering in these individuals and found that those in large clusters had distinct characteristics from individuals in the other two groups (Table 1), and were more likely to be male (85.8 vs 77.7% in small/medium clusters and 71.6% in non-clustered individuals; *P* < 0.001) and younger (29.4% of individuals < 25 years of age vs. 20.7% and 18.6%; *P* < 0.001); have RHI status (33.4% vs 30.9% and 22.8%; *P* < 0.001); report MSM contact as their main risk behavior (94.5% vs 85.4% and 84.2%; *P* < 0.001); and have a high CD4 + cell count (18% with ≥ 500 cells/μl vs 14% and 11.6%; *P* < 0.001). No significant differences in viral load were observed between groups. Other factors that increased the probability of clustering were Han ethnicity, single marital status, and residence in cities in Liaoning other than Shenyang (*P* < 0.001).

**TABLE 1:** Comparison of characteristics of HIV-1 infected individuals by cluster inclusion and cluster size.

			In cluster (*n* = 788)		
	Total (*n* = 2087)	Not in Cluster (*n* = 1299)	Small/medium clusters (2–9 members) (*n* = 444)	Large clusters (≥ 10 members) (*n* = 344)	*P*-value*

	***N*(%)**	***N*(%)**	***N*(%)**	***N*(%)**	
**Diagnosis Period**					<0.001*
2008–2010	362(17.3)	173(13.3)	88(19.8)	101(29.4)	
2011–2013	584(28)	370(28.5)	104(23.4)	110(32)	
2014–2016	1141(54.7)	756(58.2)	252(56.8)	133(38.7)	
**Sex**					<0.001*
Male	1570(75.2)	930(71.6)	345(77.7)	295(85.8)	
Female	93(4.5)	72(5.5)	17(3.8)	4(1.2)	
Unknown	424(20.3)	297(22.9)	82(18.5)	45(13.1)	
**Risk group**					<0.001*
MSM	1798(86.2)	1094(84.2)	379(85.4)	325(94.5)	
HETERO	268(12.8)	190(14.6)	59(13.3)	19(5.5)	
IDU	21(1)	15(1.2)	6(1.4)	0(0)	
**Age at diagnosis, years**					<0.001*
<25	434(20.8)	241(18.6)	92(20.7)	101(29.4)	
25–35	605(29)	342(26.3)	143(32.2)	120(34.9)	
35–45	313(15)	207(15.9)	67(15.1)	39(11.3)	
>45	305(14.6)	205(15.8)	60(13.5)	40(11.6)	
Unknown	430(20.6)	304(23.4)	82(18.5)	44(12.8)	
**Ethnic Group**					0.03*
Han	1444(69.2)	875(67.4)	318(71.6)	251(73.0)	
Manchu	148(7.1)	80(6.2)	28(6.3)	40(11.6)	
Other	68(3.3)	46(3.5)	14(3.2)	8(2.3)	
Unknown	427(20.5)	298(22.9)	84(18.9)	45(13.1)	
**Recident city**					<0.001*
Shenyang, Liaoning	1528(73.2)	939(72.3)	331(74.5)	258(75.0)	
Other cities in Liaoning	113(5.4)	50(3.8)	25(5.6)	38(11.0)	
Other province	13(0.6)	7(0.5)	4(0.9)	2(0.6)	
Unknown	433(20.7)	303(23.3)	84(18.9)	46(13.4)	
**Marital Status**					0.011*
Single	1019(48.8)	588(45.3)	216(48.6)	215(62.5)	
Married	432(20.7)	279(21.5)	98(22.1)	55(16.0)	
Divorced/Widower	202(9.7)	130(10.0)	46(10.4)	26(7.6)	
Unknown	434(20.8)	302(23.2)	84(18.9)	48(14.0)	
**Education**					0.494
College or above	744(35.6)	434(33.4)	166(37.4)	144(41.9)	
High school or below	833(39.9)	515(39.6)	176(39.6)	142(41.3)	
Unknown	510(24.4)	350(26.9)	102(23.0)	58(16.9)	
**Occupation**					0.012*
Cadre staff	359(17.2)	190(14.6)	86(19.4)	83(24.1)	
Individual business	165(7.9)	90(6.9)	41(9.2)	34(9.9)	
Workers	488(23.4)	161(12.4)	57(12.8)	57(16.6)	
Students	316(15.1)	68(5.2)	28(6.3)	24(7.0)	
Housework, Unemployment and others	428(20.5)	221(17.0)	68(15.3)	39(11.3)	
Farmers	26(1.2)	19(1.5)	4(0.9)	3(0.9)	
Other or Unknown	305(14.6)	550(42.3)	160(36.0)	104(30.2)	
**RHI or CHI**					<0.001*
RHI	548(26.3)	296(22.8)	137(30.9)	115(33.4)	
CHI	1539(73.7)	1003(77.2)	307(69.1)	229(66.6)	
**CD4 cells count/mm3**					<0.001*
≤ 200	615(29.5)	437(33.6)	103(23.2)	75(21.8)	
200–350	721(34.5)	433(33.3)	167(37.6)	121(35.2)	
350–500	425(20.4)	248(19.1)	103(23.2)	74(21.5)	
≥ 500	275(13.2)	151(11.6)	62(14)	62(18)	
Unknown	51(2.4)	30(2.3)	9(2)	12(3.5)	
**Viral loads(log10)**					0.29
≤ 4	348(16.7)	217(16.7)	70(15.8)	61(17.7)	
4–5	1066(51.1)	674(51.9)	224(50.5)	168(48.8)	
>5	572(27.4)	347(26.7)	134(30.2)	91(26.5)	
Unknown	101(4.8)	61(4.7)	16(3.6)	24(7.0)	
**The years between diagnosing and ART**					<0.001*
≤ 0.5	1307(62.6)	857(66)	274(61.7)	176(51.2)	
0.5–2	206(9.9)	121(9.3)	48(10.8)	37(10.8)	
>2	209(10)	97(7.5)	50(11.3)	62(18)	
ART naïve/lost	365(17.5)	224(17.2)	72(16.2)	69(20.1)	

*P < 0.05 was considered statistically significant; groups were compared with the Pearson’s chi-squared statistical test and Fisher’s exact test. ART, antiretroviral therapy; CHI, chronic HIV infection; Hetero, heterosexual; HIV, human immunodeficiency virus; IDU, injection drug user; MSM, men who have sex with men; RHI, recent HIV infection.

### Progressive Decline in the Proportion of Large Clusters Over Time

**FIGURE 2:**
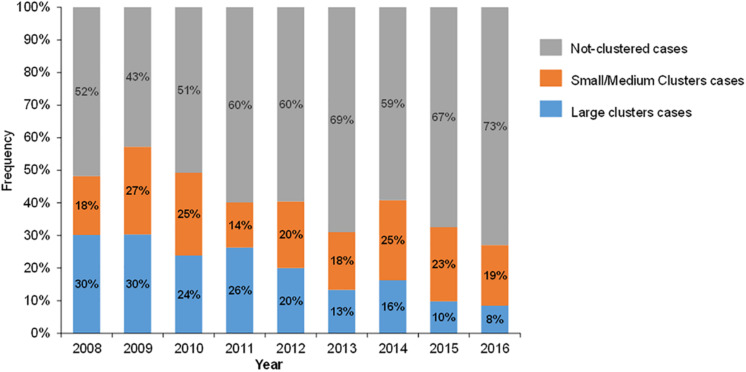
Composition of newly diagnosed HIV-1–infected cases. Blue, orange, and gray columns represent cases belonging to large clusters (≥ 10 cases), small/medium clusters (2–9 cases), and cases not in a cluster, respectively.

**FIGURE 3:**
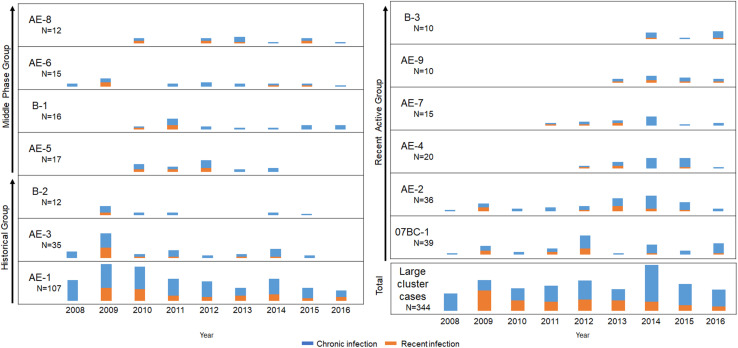
Expanding dynamics of the three groups including 13 local large clusters (≥ 10 cases) from 2008 to 2016. Years are shown along the *x*-axis and the number of new diagnoses for each cluster or all large clusters (≥ 10 cases) is shown along the *y*-axis. Orange and blue columns indicate recent and chronic HIV infection, respectively.

Individuals in large clusters (≥ 10 cases) tended to be diagnosed earlier (2008–2010) than those who were not in a cluster (29.4% vs 13.3%, *P* < 0.001) (Table 1). Further we evaluated the contribution of large clusters to the local HIV epidemic over time (Figure 2). The proportion of individuals in large clusters gradually declined from 30% in 2008 to 8% in 2016. Importantly, the proportion of individuals with RHI in large clusters also decreased from 66% in 2009 to 20% in 2016 (Figure 3), with the number of RHI cases decreasing from 29 to 6 during that period. In contrast, the percentage of non-clustered individuals increased steadily from 52% in 2008 to 73% in 2016, while no substantial changes were observed in small/medium clusters (18% in 2008 and 19% in 2016).

### Expansion History and Dynamics of Large Clusters in the Period of 2008–2016

**FIGURE 4:**
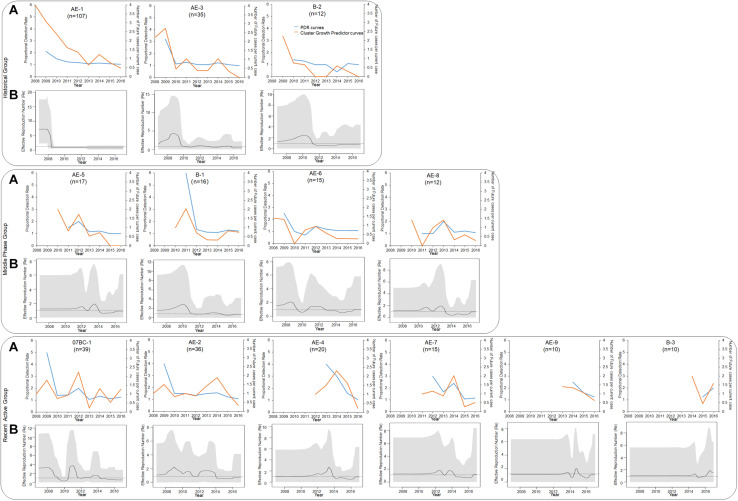
PDR trends, cluster growth predictor trends, and *R*_*e*_ of 13 large clusters (≥ 10 cases). **(A)** PDR trends (blue line), cluster growth predictor trends (orange line) **(A)**, and R_*e*_ number (secondary infections per infected person) **(B)** during the period of 2008–2016. *R*_*e*_ was inferred from the birth–death skyline plot. Thick solid lines represent the estimated mean, and 95% highest posterior density credible regions are shown as gray areas.

To clarify the dynamics of the 13 large clusters (≥ 10 cases), we analyzed the expansion history of them during the period of 2008–2016 (Figure 3). These clusters were roughly divided into historical (2008–2010), middle-phase (2011–2013), and recently active (2014–2016) according to their period of most rapid expansion (defined as > 45% of the final cluster size reached by the end of 2016). For example, the cluster AE-1 within which 48% cases were diagnosed between 2008 and 2010 belong to “Historical Group”. PDR, cluster growth predictor, and R_*e*_ were retrospectively calculated to evaluate the expansion speed of each large cluster. Although the shape of the curves varied, the three parameters confirmed that transmission of most large clusters declined during the study period (Figures 4A,B).

For the historical group (clusters AE-1, AE-3, and B-2), both newly diagnosed (48%, 51%, and 58%) and RHI (48%, 75%, and 100%) cases were concentrated between 2008 and 2010 (Figures 3, 4). Similarly, both PDR and cluster growth predictor declined, with slight fluctuations. The peak of the R_*e*_ curves for AE-1, AE-3, and B-2 occurred in 2008, 2009, and 2010, respectively. The R_*e*_ of all of three clusters declined and remained at 1 for > 5 years, implying that the clusters were historical and have receded in recent years.

The middle-phase group included clusters AE-5, B-1, AE-6, and AE-8, which had more cases diagnosed between 2011 and 2013 than at any other time (59%, 50%, 47%, and 50%, respectively); three of the clusters (B-1, AE-5, and AE-8) had even higher proportions of RHI cases diagnosed during this period (75%, 71.4%, and 42.9%, respectively) (Figures 3, 4). These data were consistent with the trends observed for PDR and cluster growth predictor and demonstrated that all clusters underwent rapid expansion in 2011–2013 and declined thereafter. The R_*e*_ of clusters B-1 and AE-6 were relatively high before 2010, while AE-5 and AE-8 were expanding during 2012–2014 and 2010–2014, respectively. However, all four clusters showed a stable R_*e*_ of 1 in recent years.

The recently active group (clusters 07BC-1, AE-2, AE-4, AE-7, AE-9, and B-3) had more newly diagnosed cases (> 45%) in 2014–2016 than at any other time (Figures 3, 4). AE-9 and B-3 were recently emerged clusters that appeared in 2013 and 2014, respectively. The PDR of 5 of the 6 recently active clusters decreased or remained at a constant low level; the exception was cluster B-3, for which PDR increased from 1.25 in 2015 to 2 in 2016. Similarly, the cluster growth curves predictor of AE-2, AE-4, and AE-9 fluctuated but declined toward the end of the study period, while the curves for 07BC-1, AE-7, and B-3 showed an upward trend. According to the birth–death model, cluster 07BC-1 and AE-7 declined and remained at 1 in 2016, as well as cluster AE-2, AE-4 and AE-9. On the contrary, the R_*e*_ of B-3 increased between 2014 and 2016 and reached 2.676 in 2016. Of the cluster with upward curves (Supplementary Table S2), cases in B-3 tended to be younger (mean age, 26.6 years) and had a higher proportion of local MSM (100%).

### Reduced Ongoing Expansion of Large Clusters Coincident With Earlier Initiation of ART

**FIGURE 5:**
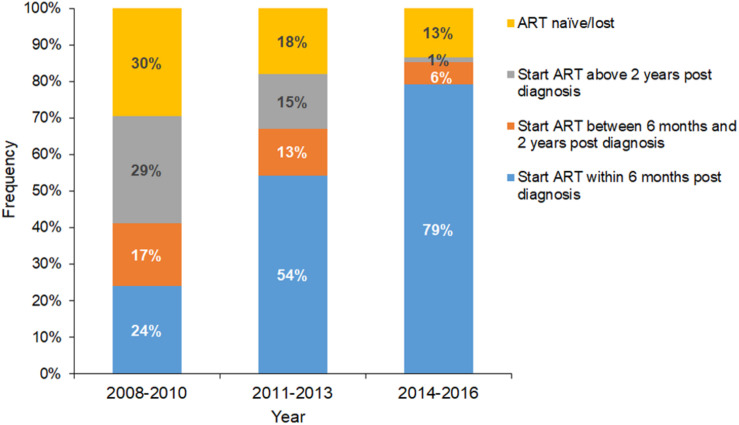
Distribution and composition of the time lag between HIV infection diagnosis and ART initiation. Blue, orange, gray, and yellow columns represent ART initiation within 6 months of diagnosis, ART initiation between 0.5 and 2 years post diagnosis, ART initiation > 2 years post diagnosis, and ART naïve/lost, respectively.

Since the establishment of this long-term cohorts in Shenyang in 2008, the standards of ART for HIV-infected patients in China have incrementally improved. Our data showed that the proportion of cases who initiated ART within 6 months after diagnosis increased from 24% in 2008–2010 to 54% in 2011–2013 and 79% in 2014–2016 (Figure 5). In contrast, the proportion of cases who initiated ART > 2 years after diagnosis decreased from 29% in 2008–2010 to 15% in 2011–2013 and 1% in 2014–2016. Meanwhile, the proportion of patients starting ART between 0.5 and 2 years post diagnosis also decreased (from 17% in 2008–2010 to 6% in 2014–2016), as did the proportion without medical care (from 30% in 2008–2010 to 13% in 2014–2016). It is worth noting that large clusters had a higher percentage of patients who delayed ART or did not receive treatment (38.1%) compared to non-clustered cases (24.7%) and small/medium clusters (27.5%) (*P* < 0.0001; Table 1), and were mainly concentrated in the period of 2008–2010 (data not shown).

## Discussion

In this study we retrospectively reconstructed the molecular networks of three main HIV-1 subtypes among patients who were newly diagnosed with HIV-1 infection in Shenyang in 2008–2016. Our results show that the expansion of large clusters was progressively controlled, coinciding with and supporting the effectiveness of strengthened early ART and continuous regular interventions.

### Threshold Selection in an Area With Multiple HIV-1 Subtypes

The threshold is a key factor in molecular network construction (Hassan et al., 2017). Studies on HIV evolution-which have mainly focused on HIV-1 subtype B- have supported threshold selection and molecular network-guided applications; 1.5% was selected as the optimal threshold based on a rate of evolution of 1% every 10 years for the pol gene (Smith et al., 2009; Hightower et al., 2013). Nearly all molecular network studies on non-B HIV have used the subtype B threshold (Bon et al., 2010; Parczewski et al., 2012; Rose et al., 2017; Fabeni et al., 2019), however, the threshold for subtype B are also appropriate for non-B viral strains have not been fully explored (Han et al., 2020).

Unlike areas with “a single subtype” of HIV- e.g., Western and central Europe and North America (83.3% subtype B); and Southern Africa (98.8% subtype C) (Hemelaar et al., 2019)-there were three subtypes accounting for up to 94% of cases in Shenyang. The prevalence of CRF01_AE and subtype B decreased by around 20% (from 91.9 to 73.6%) between 2008 and 2016, whereas that of CRF07_BC and other CRFs or URFs increased. The few network studies that have been carried out in areas with multiple HIV subtypes and have typically used one single genetic threshold (Hoenigl et al., 2016a; Stecher et al., 2018). Rates of HIV-1 evolution vary across subtypes (Patino-Galindo and Gonzalez-Candelas, 2017; Bbosa et al., 2019a), and no previous studies have focused on HIV evolution or threshold selection for our local epidemic strains. We selected an optimal threshold according to the principle outlined in a previous study (Wertheim et al., 2017) in order to identify the maximum number of clusters in the genetic network. Above the threshold, clusters began to coalesce and the network lost resolution (Supplementary Figure S3). This principle has been used in several recent studies (Chaillon et al., 2019; Ragonnet-Cronin et al., 2019; Zai et al., 2020). We used separate optimal thresholds for CRF01_AE, CRF07_BC, and subtype B, rather than one threshold for multiple subtypes.

### Control of Local Large Clusters Has Coincided With Strengthened Early ART

We used HIV molecular networks to identify closely related transmission events; a cluster was formed if the pairwise genetic distance of any two sequences was less than the optimal threshold. A large cluster was treated as a large-scale spreading event in which already highly connected individuals made proportionally more contacts over time (Ragonnet-Cronin et al., 2016). In our study, more individuals with delayed ART or without ART in large clusters were diagnosed in 2008–2010 than in the other two phases of the study, and were likely a source of infection that contributed to the increase in the number of new diagnoses in subsequent years. However, the transmission of most large clusters showed a declining trend at the end of the study, as evidenced by the reductions in the number of new cases in each cluster and in the three parameters of cluster growth (Figures 3, 4). These results suggest that the contribution of large clusters to the local HIV epidemic decreased over time and that these clusters may not be the main driving force of future HIV epidemics.

Moreover, transmissibility is highest in the early stage of HIV infection and plays an important role in ongoing transmission (Brenner et al., 2007; Powers et al., 2011; Marzel et al., 2016). Therefore, clusters with more RHIs are thought to be more active and should be prioritized for intervention. In our study, the rate of RHIs detected by HIV-1 LAg-Avidity EIA or determined from seroconversion records decreased over time in 12 of the 13 large clusters (with cluster B-3 being the exception) (Figure 3), providing further evidence of HIV large transmission clusters declined.

A series of major policies and regulations have been promulgated by the Chinese government to control the spread of HIV. Since the initiation of the National Free Antiretroviral Treatment Program (Zhang et al., 2007; Zhang et al., 2009; Cao et al., 2020) and “Four Frees and One Care” (Sun et al., 2010) program in 2002 and 2003, respectively, HIV testing and access to care have markedly improved, and the free ART program has been rapidly scaled up (Wu et al., 2017; Cao et al., 2020). In 2008, a large prospective cohort of HIV-1–seronegative high-risk individuals were established in Shenyang that received continuous education on HIV and free HIV testing and counseling, and were regularly followed up. Nonetheless, the most significant change during the study period was the improvement of treatment standards and the expansion of treatment coverage, with the criterion for ART initiation changing from CD4 + T cell counts ≤ 350 cells/μl in 2008 to ≤ 500 cells/μl in 2014, with immediate ART now recommended for all patients after a diagnosis of HIV infection. According to our data, early ART (initiated within 6 months after diagnosis) increased from 24 to 79% between 2008 and 2016 (Figure 5), which coincided with decreases in both the ongoing transmission of large clusters and the number of newly diagnosed RHI cases in large clusters, indicating the control of HIV transmission and the effectiveness of preventative treatments (Figures 4, 5). Similarly, a study from Belgium demonstrated that the expansion of an outbreak cluster (subtype F1 outbreak among MSM) was controlled by 2012 (*R*_*e*_ < 1), coinciding with a decrease in rates of delayed ART initiation as a result of implementation of the immediate ART initiation policy (Vinken et al., 2019).

### Significance of Large Clusters and Other Groups Contributing to the Local Epidemic

In a previous study by the U.S. National HIV Surveillance System, the transmission rates of 11 large clusters were 11 times higher than the national average and were targeted for intervention (France et al., 2018; Oster et al., 2018a,b). Another study conducted in Canada showed that large cluster cases were progressively increasing, contributing to ≥ 40% of ongoing local HIV-1 transmission events (Brenner et al., 2017). Our results also showed that individuals in large clusters were younger, single, had high CD4 counts, were recent infected, were MSM and had delayed/no ART compared to individuals in other groups (Table 1). Thus, individuals in large clusters may have higher risk of further transmission in later years compared to those in small/medium clusters and the non-clustered group (Oster et al., 2018a). We therefore focused on the expansion of large clusters and speculate that their declining contributions over time indicate the progressive control of the local epidemic of main HIV-1 strains in the studied area.

During the study period, the number of newly diagnosed cases in Shenyang was still increasing. HIV infection has a long asymptomatic period, and an infected case can be diagnosed at any time during that period. Thus, the number of newly diagnosed cases was largely affected by testing and did not accurately reflect the severity of the HIV epidemic. RHIs were detected with the HIV-1Lag avidity EIA, and our results showed that the incidence of RHIs was stable during the study period, while that of CHI increased markedly in 2014 and declined slightly thereafter, which we think was due to the start of medical referral from other sites to the study center for treatment. Indeed, the sampling depth of our study increase from 39% in 2013 to 54% in 2014. Moreover, a large number of patients with CHIs who had actually been infected in the past were diagnosed during this period by continuous testing. Given these conditions, new strategies are needed to evaluate the dynamics of HIV epidemics and the effectiveness of interventions.

Non-clustering refers to an absence of links to any cases or clusters (Bbosa et al., 2019b). The non-clustered cases may be: a) the referred patients from other surveillance sites; b) inflow of cases who infected HIV at other cities; c) CHIs with long-term within-host evolution history; and d) recombinant of intra-lineages strains leading the genetic distance gap from other pure sequences. In fact, non-clustering is one of the most overlooked topics in the current literatures; how to manage the growing non-clustered population is an important question that should be addressed.

### Perspectives and Recommendations

Our molecular cluster analysis provided evidence that large clusters in Shenyang have been gradually controlled, while highlighting the need to identify individuals who should be prioritized for intervention based on their association with high-risk clusters. To achieve this goal, we recommend the followings. Firstly, both the percentage of RHI cases in recent years and potential for future growth of a cluster should be considered. Cluster B-3 in our study was a rapidly expanding cluster of subtype B that showed increasing PDR and cluster growth predictor curves and *R*_*e*_ > 1. It was also a newly emerged cluster with 2 RHIs, which met the criteria of an active cluster for prioritized intervention according to China Center for Disease Control and Prevention guidelines (China-CDC, 2019) Cluster B-3 was shown to have undergone an expansion between 2014 and 2016, and is expected to grow further in coming years. Secondly, interventions should aim to control viral replication in rapidly expanding clusters, particularly those with cases in the early stages of infection. Thirdly, partner services as well as increased testing and intervention options should be provided to persons associated with high-risk clusters. Lastly, additional specific interventions should be considered depending on the characteristics of the high-risk cluster (e.g., cluster B-3), and individuals with the same characteristics should be closely monitored in terms of HIV status and referred for PrEP even if they are HIV-1–seronegative.

In this study, complementary epidemiologic and phylodynamic analyses were used to evaluate the growth tendency of large clusters and identify priorities for intervention. Both PDR and cluster growth predictor are simplified algorithms (Wertheim et al., 2018; Dennis et al., 2020) based on epidemiologic data (number of cases diagnosed each year), and can be easily determined. The curves for both parameters have similar shapes but the PDR curve is more stable, whereas that of cluster growth predictor fluctuates and reveals small changes, especially in larger clusters. For example, in our study a large number of new cases were referred from other surveillance sites to the study center for treatment since 2014; therefore, a peak was observed in the cluster growth predictor curve of many clusters in 2014 (orange lines in Figure 4), whereas no corresponding change was observed in the PDR curves (blue lines in Figure 4). R_*e*_, the third phylodynamic parameter that is based on sequence diversity, reflects the efficiency with which an infectious agent is transmitted and is frequently used to model infection dynamics (Novitsky et al., 2015; Ragonnet-Cronin et al., 2018; Jovanovic et al., 2019; Lorenzin et al., 2019; Vasylyeva et al., 2019; Vinken et al., 2019; Zai et al., 2020). We found that R_*e*_ was accurate and reliable. However, unlike the PDR independent of data volume, R_*e*_ was unsuitable for small clusters. Thus, the three parameters can be applied in different ways to real-time monitoring of molecular networks for the identification of rapidly expanding clusters: PDR is more suitable for smaller clusters while cluster growth predictor is better for larger ones, and R_*e*_ can be used for final verification of mid-sized or large clusters.

### Limitations

The assessment of HIV molecular clusters is influenced by sampling depth. In our study, the average sampling rate of 45% may have limited the ability to detect transmission clusters. Additionally, recombination events between diverse HIV strains made it challenging to distinguish clusters, as recombinants are typically removed from analytic datasets (Grabowski et al., 2018). In this study, we analyzed only three major subtypes of HIV-1 (CRF01_AE, CRF07_BC, and subtype B); other CRFs and URFs (6%) were not included because the optimal thresholds could not be determined given the diverse origins and rates of evolution of the different strains.

## Conclusion

In summary, the results of this study show that large HIV transmission clusters declined in Shenyang between 2008 and 2016, coinciding with the implementation of early ART and continuous regular interventions and confirming the effectiveness of these strategies. We also demonstrated that molecular network analyses can be used to evaluate the efficacy of interventions in areas of epidemic with a complex HIV profile, which can guide the implementation of targeted interventions.

## Data Availability Statement

The datasets presented in this study can be found in online repositories. The names of the repository/repositories and accession number(s) can be found below: https://www.ncbi.nlm.nih.gov/genbank/, MT368043-369927; https://www.ncbi.nlm.nih.gov/genbank/, MT336755-336776.

## Author Contributions

HS and XH conceived and designed the study. BZ, ML, ZW, and YQ performed experimental work, BZ, and HD for the data collection. ML performed molecular and phylodynamic analyses. ML and XH wrote the first draft. All authors read and approved the final manuscript.

## Conflict of Interest

The authors declare that the research was conducted in the absence of any commercial or financial relationships that could be construed as a potential conflict of interest.

## Footnotes

1 www.hiv.lanl.gov

2 www.hivtrace.org

## Supplementary Material

Supplementary Figure 1 Maximum-likelihood phylogenetic tree of HIV-1 pol sequences from 2221 infected individuals in Shenyang, China. The maximum likelihood (ML) tree was reconstructed using the partial pol sequences under GTR + I + G sites substitution model. The ML tree is rooted with subtype N sequence as outgroup. The support values of the nodes was estimated with the approximate likelihood-ratio (aLRT) SH-like test. CRF01_AE sequences are in red, CRF07_BC sequences are in blue and subtype B are in green. Other CRFs, URFs and their references are in black.

Supplementary Figure 2 Distribution of HIV-1 subtypes in newly diagnosed infected individuals between January 2008 and December 2016. Columns represent the number of newly diagnosed HIV-infected individuals annually; blue represents CRF01_AE, orange represents CRF07_BC, gray represents subtype B, and yellow represents other CRFs or URFs. The pie chart shows the proportion of each subtype across all individuals.

Supplementary Figure 3 Number of nodes and transmission clusters as a function of Tamura–Nei 93 distance thresholds. **(A)** CRF01_AE. **(B)** CRF07_BC. **(C)** Subtype B. Blue and orange lines represent the number of clusters and nodes, respectively

Supplementary Table 1 Characteristics of Molecular Networks at Optimal Genetic Threshold of CRF01_AE, CRF07_BC, and subtype B.

Supplementary Table 2 Demographic Characteristics of the 13 Large Transmission Clusters.
