# Supplementary figures and images for: Dynamics of HIV-1 Molecular Networks Reveal Effective Control of Large Transmission Clusters in an Area Affected by an Epidemic of Multiple HIV Subtypes
Source: Front Microbiol. 2020 Nov 13;11:604993. doi: 10.3389/fmicb.2020.604993 (PMC7691493; doi:10.3389/fmicb.2020.604993)

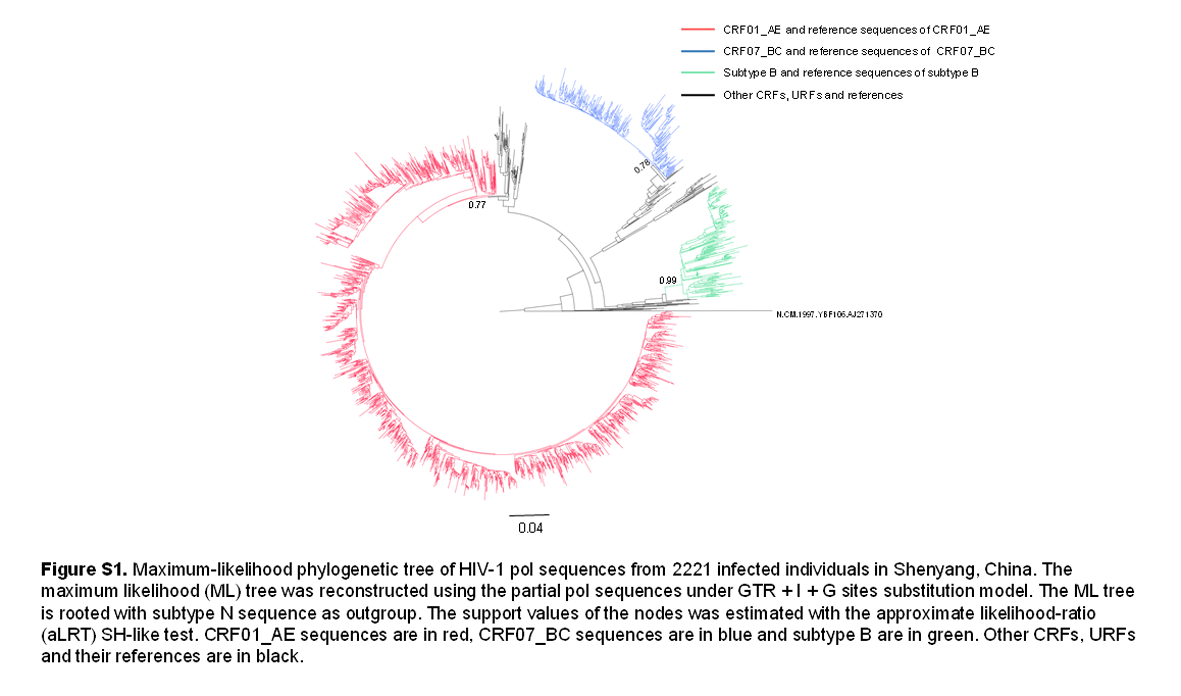

Supplement: Supplementary Figure 1 — Maximum-likelihood phylogenetic tree of HIV-1 pol sequences from 2221 infected individuals in Shenyang, China. The maximum likelihood (ML) tree was reconstructed using the partial pol sequences under GTR + I + G sites substitution model. The ML tree is rooted with subtype N sequence as outgroup. The support values of the nodes was estimated with the approximate likelihood-ratio (aLRT) SH-like test. CRF01_AE sequences are in red, CRF07_BC sequences are in blue and subtype B are in green. Other CRFs, URFs and their references are in black. [file Image_1.TIF]

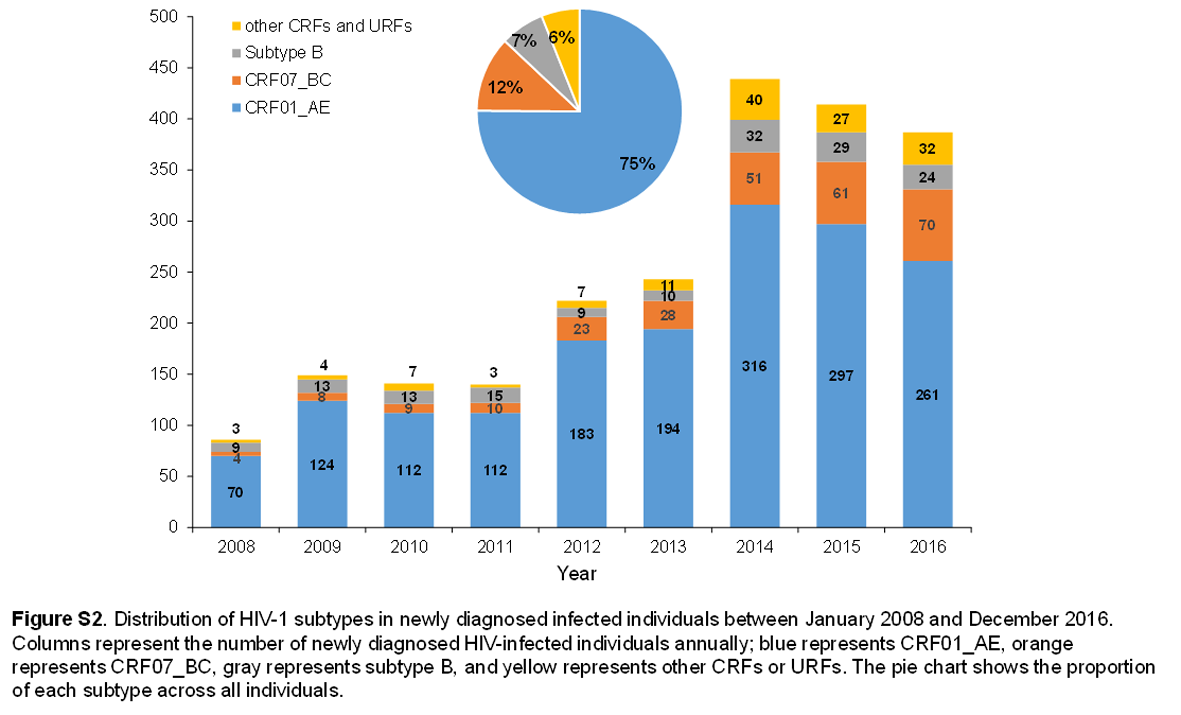

Supplement: Supplementary Figure 2 — Distribution of HIV-1 subtypes in newly diagnosed infected individuals between January 2008 and December 2016. Columns represent the number of newly diagnosed HIV-infected individuals annually; blue represents CRF01_AE, orange represents CRF07_BC, gray represents subtype B, and yellow represents other CRFs or URFs. The pie chart shows the proportion of each subtype across all individuals. [file Image_2.TIF]

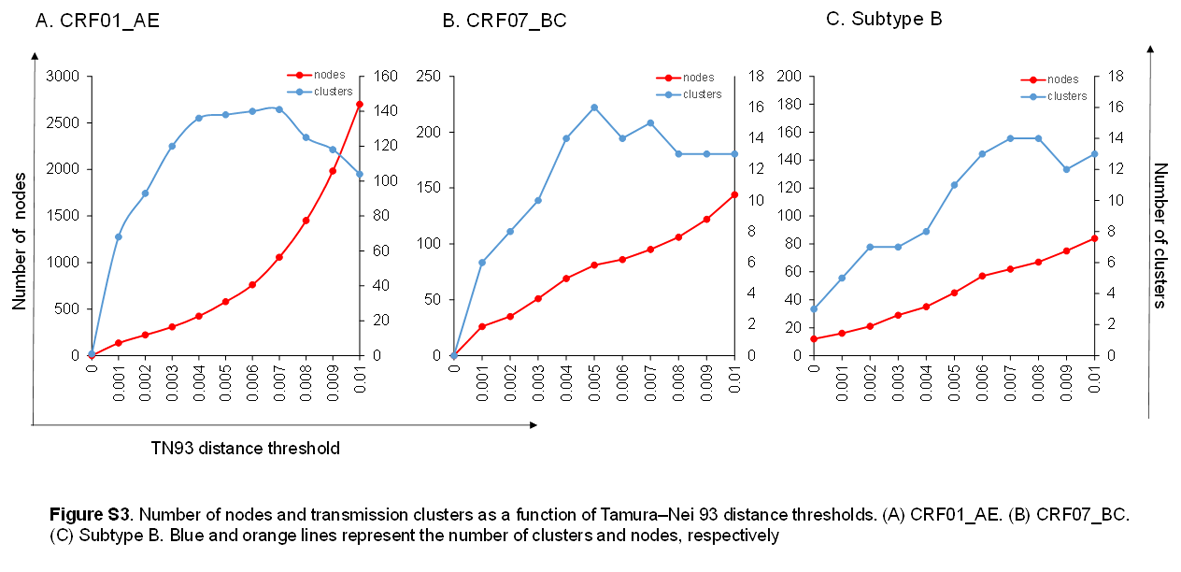

Supplement: Supplementary Figure 3 — Number of nodes and transmission clusters as a function of Tamura–Nei 93 distance thresholds. (A) CRF01_AE. (B) CRF07_BC. (C) Subtype B. Blue and orange lines represent the number of clusters and nodes, respectively [file Image_3.TIF]
