# Supplementary material for: Dynamics of HIV-1 Molecular Networks Reveal Effective Control of Large Transmission Clusters in an Area Affected by an Epidemic of Multiple HIV Subtypes
Source: Front Microbiol. 2020 Nov 13;11:604993. doi: 10.3389/fmicb.2020.604993 (PMC7691493; doi:10.3389/fmicb.2020.604993)
Supplement: Supplementary Table 1 — Characteristics of Molecular Networks at Optimal Genetic Threshold of CRF01_AE, CRF07_BC, and subtype B. [file Table_1.DOCX]

Table S1. Characteristics of Molecular Networks at Optimal Genetic Threshold of CRF01_AE, CRF07_BC and subtype B.

|  | **Optimal Genetic Threshold** | **Clusters** | **Nodes** | **Cluster Rate** |
| --- | --- | --- | --- | --- |
| CRF01_AE | 0.005 | 138 | 645 | 38.6% |
| CRF07_BC | 0.005 | 16 | 81 | 30.7% |
| Subtype B | 0.007 | 14 | 62 | 40.3% |
