# Supplementary material for: Dynamics of HIV-1 Molecular Networks Reveal Effective Control of Large Transmission Clusters in an Area Affected by an Epidemic of Multiple HIV Subtypes
Source: Front Microbiol. 2020 Nov 13;11:604993. doi: 10.3389/fmicb.2020.604993 (PMC7691493; doi:10.3389/fmicb.2020.604993)
Supplement: Supplementary Table 2 — Demographic Characteristics of the 13 Large Transmission Clusters. [file Table_2.DOCX]

Table S2. Demographic Characteristics of the 13 Large Transmission Clusters.

| **Large Cluster** | **Size** | **Recent%** | **Sampling**  **Year** | **Age at Diagnosis**  **Median[Range]** | | **MSM%** | **Male%** | **Residence Shenyang%** | **Han Ethnic%** | **Single%** | **College/above%** | **Genetic Distance**  **Median[IQR]** |
| --- | --- | --- | --- | --- | --- | --- | --- | --- | --- | --- | --- | --- |
| AE-1 | 107 | 37.40% | 2008-2016 | 31[17-68] | 92.50% | | 99.00% | 86.90% | 83.20% | 58.40% | 41.70% | 0.0040 [0.0020 - 0.0049] |
| 07BC-1 | 39 | 30.80% | 2008-2016 | 27[19-67] | 97.40% | | 96.60% | 100.00% | 86.20% | 82.10% | 69.20% | 0.0030 [0.0020 - 0.0040] |
| AE-2 | 36 | 30.60% | 2008-2016 | 29[18-49] | 97.20% | | 100.00% | 84.80% | 87.90% | 81.80% | 43.80% | 0.0040 [0.0020 - 0.0049] |
| AE-3 | 35 | 34.30% | 2008-2015 | 26[18-55] | 97.10% | | 100.00% | 57.10% | 88.60% | 84.80% | 50.00% | 0.0040 [0.0030 - 0.0049] |
| AE-4 | 20 | 20.00% | 2012-2016 | 31[21-48] | 85.00% | | 100.00% | 100.00% | 82.40% | 70.60% | 81.30% | 0.0040 [0.0030 - 0.0050] |
| AE-5 | 17 | 41.20% | 2010-2014 | 30[19-47] | 100.00% | | 100.00% | 80.00% | 73.30% | 66.70% | 42.90% | 0.0040 [0.0020 - 0.0049] |
| B-1 | 16 | 25.00% | 2010-2016 | 29[22-35] | 87.50% | | 92.30% | 100.00% | 76.90% | 76.90% | 61.60% | 0.0049 [0.0049 - 0.0059] |
| AE-6 | 15 | 33.30% | 2008-2016 | 24[22-47] | 100.00% | | 100.00% | 85.70% | 85.70% | 78.60% | 57.20% | 0.0020 [0.00099 - 0.0030] |
| AE-7 | 15 | 26.70% | 2011-2016 | 23[18-32] | 100.00% | | 100.00% | 91.70% | 81.80% | 100.00% | 45.50% | 0.0040 [0.0040 - 0.0049] |
| B-2 | 12 | 16.70% | 2009-2015 | 28[21-35] | 100.00% | | 100.00% | 90.90% | 90.90% | 100.00% | 60.00% | 0.0034 [0.0015 - 0.0041] |
| AE-8 | 12 | 58.30% | 2010-2016 | 43[28-47] | 83.30% | | 87.50% | 100.00% | 87.50% | 37.50% | 25.00% | 0.0030 [0.00099 - 0.0040] |
| AE-9 | 10 | 50.00% | 2013-2016 | 25[16-42] | 90.00% | | 100.00% | 100.00% | 85.70% | 85.70% | 71.40% | 0.0030 [0.0020 - 0.0045] |
| B-3 | 10 | 20.00% | 2014-2016 | 27[22-34] | 100.00% | | 100.00% | 100.00% | 60.00% | 80.00% | 40.00% | 0.00097 [0.0 - 0.0049] |
